# Yet More “Weeds” in the Garden: Fungal Novelties from Nests of Leaf-Cutting Ants

**DOI:** 10.1371/journal.pone.0082265

**Published:** 2013-12-20

**Authors:** Juliana O. Augustin, Johannes Z. Groenewald, Robson J. Nascimento, Eduardo S. G. Mizubuti, Robert W. Barreto, Simon L. Elliot, Harry C. Evans

**Affiliations:** 1 Departamento de Entomologia, Universidade Federal de Viçosa, Viçosa, Minas Gerais, Brazil; 2 Centraalbureau voor Schimmelcultures–Fungal Biodiversity Centre, Utrecht, The Netherlands; 3 Departamento de Fitopatologia, Universidade Federal de Viçosa, Viçosa, Minas Gerais, Brazil; 4 Centre for Agriculture and Biosciences International, Egham, Surrey, United Kingdom; University of California Riverside, United States of America

## Abstract

**Background:**

Symbiotic relationships modulate the evolution of living organisms in all levels of biological organization. A notable example of symbiosis is that of attine ants (*Attini*; *Formicidae*: *Hymenoptera*) and their fungal cultivars (*Lepiotaceae* and *Pterulaceae*; *Agaricales*: *Basidiomycota*). In recent years, this mutualism has emerged as a model system for studying coevolution, speciation, and multitrophic interactions. Ubiquitous in this ant-fungal symbiosis is the “weedy” fungus *Escovopsis* (*Hypocreales*: *Ascomycota*), known only as a mycoparasite of attine fungal gardens. Despite interest in its biology, ecology and molecular phylogeny—noting, especially, the high genetic diversity encountered—which has led to a steady flow of publications over the past decade, only two species of *Escovopsis* have formally been described.

**Methods and Results:**

We sampled from fungal gardens and garden waste (middens) of nests of the leaf-cutting ant genus *Acromyrmex* in a remnant of subtropical Atlantic rainforest in Minas Gerais, Brazil. In culture, distinct morphotypes of *Escovopsis* sensu lato were recognized. Using both morphological and molecular analyses, three new species of *Escovopsis* were identified. These are described and illustrated herein—*E. lentecrescens*, *E. microspora*, and *E. moelleri*—together with a re-description of the genus and the type species, *E. weberi*. The new genus *Escovopsioides* is erected for a fourth morphotype. We identify, for the first time, a mechanism for horizontal transmission via middens.

**Conclusions:**

The present study makes a start at assigning names and formal descriptions to these specific fungal parasites of attine nests. Based on the results of this exploratory and geographically-restricted survey, we expect there to be many more species of the genus *Escovopsis* and its relatives associated with nests of both the lower and higher *Attini* throughout their neotropical range, as suggested in previous studies.

## Introduction

There is increasing evidence that actual and predicted levels of biodiversity in the kingdoms of life have been greatly underestimated, especially in the Kingdom Fungi [1]. One of the more recent studies has put the number of catalogued fungal species at just over 45,000, with a predicted total of around 610,000 [2]. This is at odds with the mycological records that estimate the number of described species at between 80–100,000 [3]; with predicted levels from 1.5 million [4], up to 5.1 million [1]—the latter based on high-throughput DNA sequencing. It has been suggested that this naming and cataloguing of biodiversity is akin to stamp collecting, but May [5] argues that: “To the contrary, we increasingly recognize that such knowledge is important for full understanding of the ecological and evolutionary processes…”, and, moreover, that “It also underpins ecosystem services that humanity is dependent upon”.

Currently, we are assessing the diversity of fungal parasites associated with ant societies in a fragment of the highly-threatened Atlantic rainforest in the south-eastern region of the Brazilian state of Minas Gerais. Our work, thus far, has revealed an unexpectedly high level of speciation in the zombie-ant fungus *Ophiocordyceps unilateralis* (Tul.) Petch sensu lato [6]. This has led to the suggestion that it could be a keystone taxon for understanding ecosystem functioning and fungal diversity in tropical forests [7], as well as to the hypothesis that each species of the ant tribe *Camponotini* could be parasitized by a unique species of the *O. unilateralis* clade [6], [7]. This hypothesis seems to be supported following the recent description of three new species within the *O. unilateralis* complex, each associated with a different species of carpenter ant (*Camponotus*) in Thailand [8]. Here, we look at the diversity of mycoparasites associated with the fungal gardens of leaf-cutting ants of the tribe *Attini* in the same fragment of Atlantic rainforest.

In insect societies, leaf-cutting ants are unique in their habit of harvesting fresh vegetation. This serves as a substrate for the gardens in which the ants tend their fungal cultivars, the main food source of the colonies. These fungal gardens, built in subterranean chambers, provide a stable habitat with few competitors. Such highly evolved traits have made leaf-cutting ants the dominant herbivores in the Neotropics, according to Hölldobler and Wilson [9]. In leaf-cutting ants (*Acromyrmex*, *Atta*), the mutualistic cultivars in the fungal gardens are highly derived species of the basidiomycete genus *Leucoagaricus* (*Agaricaceae*, *Agaricales*)[10]–[12]. However, the stability of this mutualism is constantly under threat from specific fungal parasites belonging to the ascomycete genus *Escovopsis* (*Hypocreales*) [13].

Although frequently found in leaf-cutting ant colonies [13], the mechanism by which this mycoparasite gains access to the nest has never been resolved. Because reproductive founding queens have not yet been discovered carrying *Escovopsis* externally, on their cuticles, or internally, in the incipient mutualistic fungal pellet that provides nourishment during the claustral founding stage of the colonies, it has been concluded that transmission of the mycoparasite between leaf-cutting ant colonies is horizontal [13], [14].

The genus *Escovopsis* was erected by Muchovej and Della Lucia in 1990 to replace *Phialocladus* Kreisel (1972), which they considered to be an invalid genus since it had been described without designating a holotype species [15], [16]. The holotype of the new genus was based on a strain recovered from the laboratory-maintained nest of an unspecified leaf-cutting ant in Minas Gerais, Brazil. The type species was named as *Escovopsis weberii* Muchovej & Della Lucia [15], but it is now listed in *Index Fungorum* as *E. weberi*, presumably because the original name was an orthographic error. The authors assumed this to be identical to *Phialocladus zsoltii* Kreisel, isolated from a fungal garden of *Atta insularis* Guérin in Cuba [16], despite differences in morphology: such as the “microconidia” of *P. zsoltii* being signficantly larger than those of *E. weberi* [15], [16]. Further to their argument that *Phialocladus* is a nomen invalidum—and that this warranted a name change of both genus and species—they stressed that the sporogenous cells are not phialidic, and, therefore, that “it is troublesome to validate the name *Phialocladus* since it would be understood from the root of the name that sporogenesis is phialidic” [15]. In addition, they failed to find the “macroconidia” included in the original diagnosis [16], and so excluded this character from the generic description.

On describing a second species in the genus—*E. aspergilloides* Seifert, Samson & Chapela—it was concluded that: “We find no reason to believe that the conidiogenous cells of *Escovopsis* species are not phialides” [17] = ‘a spore-forming cell from which the conidia are produced in basipetal succession from an open end’ [3]. The new species was duly characterized as having globose *Aspergillus*-like vesicles, rather than the clavate to cylindrical, phialide-bearing vesicles of the type species. There may be some counter claims, therefore, for resurrecting the genus *Phialocladus* which was illustrated beautifully by Carmichael et al. [18], and who included it amongst the phialiform fungi. At that time, both the biology and phylogeny of the fungus were uncertain; although it was reported to be associated with ant nests in decline and, possibly, even represented a basidiomycete anamorph [19]. Nevertheless, because *Escovopsis* is now in such common usage—especially in the many disciplines involved in studying leaf-cutting ants—the name is conserved here.

Much earlier, however, Moeller in 1893 provided the first descriptions and illustrations of both the *E. weberi* and *E. aspergilloides* vesicle forms, based on material from Brazil [19]. He did not assign formal names, but his accurate drawings were subsequently reproduced by Seifert et al. [17]. Moeller described ‘*E. weberi*’ as the dominant (“starke” or ‘strong’) conidial form associated with *Atta* nests and ‘*E. aspergilloides*’ as exclusive to colonies of the lower *Attini* genus *Apterostigma* (“Haarameisen” or ‘hairy ant’). Similarly, nearly 50 years later, Stahel and Geijskes (1941) also illustrated this distinctive but still unnamed fungal genus overgrowing a colony of *Atta sexdens* in Surinam [20]. More than two decades later, Weber (1966) again commented on the presence of this fungus in nests of “fungus-growing ants”, accompanied by a poor but distinctive illustration, and connected its presence with “abnormal circumstances” in a colony of *Trachymyrmex septentrionalis* (McCook) [21]. However, Seifert et al. later speculated that: “Species of *Escovopsis* might be important symbionts in the fungal gardens of leaf-cutting ants, but they do not represent anamorphs of the associated symbiotic basidiomycetes” [17], as suggested earlier [19]. It is strange, therefore, that despite the multiple citations and illustrations of this highly distinct and morphologically unique fungus in *Attini* nests, it was not classified taxonomically until almost 80 years after its first description [16], [19].

There has been increasing awareness about the impact of mycoparasites on nests of the *Attini*—and thus of their ecological and evolutionary significance—and, in particular, of the discovery that *Escovopsis* is a coevolved pathogen of the fungal gardens [13], representing a new clade or even a new family within the *Hypocreales* [22]. This has resulted in a steady flow of publications on the biology, ecology and molecular phylogeny of *Escovopsis* [22]–[33]; including ant defence strategies to control such mycoparasites [34]–[39]. Earlier, Currie et al. [13], using morphological characters, had identified at least eight distinct taxa of *Escovopsis* from attine nests in Panama alone, but these have never been described, and publications since then have confirmed the high genetic diversity amongst isolates of *Escovopsis* from fungal gardens [23], [32]. Despite the fact that many of these studies repeatedly emphasize that there is considerable morphological and genetic variation within the isolates, the taxonomy of the genus has not been addressed. Most studies distinguish them simply by spore or colony colour [27]. Clearly, there could be many more species throughout the geographic range of the *Attini* and each may also interact differently with their host. The present study makes a start at assigning names and formal descriptions to at least a small number of these taxa isolated from the fungal gardens—as well as from the garden waste or middens—of ants of the genus *Acromyrmex* collected during exploratory surveys in the Atlantic rainforest region of Minas Gerais, Brazil. It also highlights morphological features of the fungi that provide insights into their strategies of host exploitation, as well as their dispersal between hosts.

## Results and Discussion

**Figure 1:**
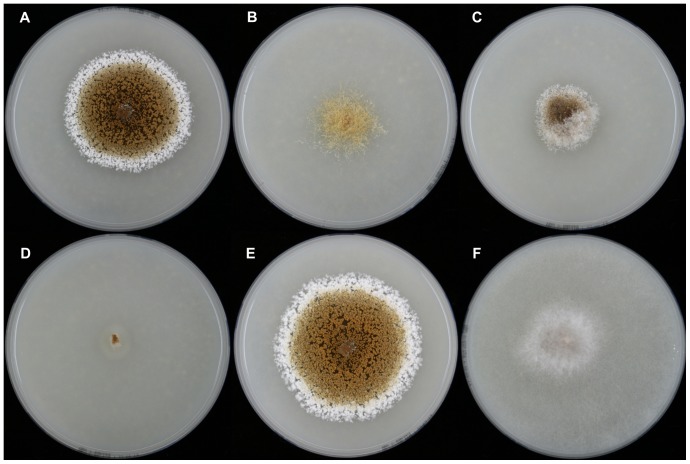
Comparison of cultural characteristics of the new taxa compared with *E. aspergilloides* and *E. weberi*. (**A**) *Escovopsis weberi* ATCC 64542, ex-type; (**B**) *Escovopsis aspergilloides* CBS 423.93, ex-type; (**C**) *Escovopsis moelleri* CBS 135748, ex-type; (**D**) *Escovopsis lentecrescens* CBS 135750, holotype; (**E**) *Escovopsis microspora* CBS 135751, ex-type; (**F**) *Escovopsioides nivea* CBS 135749, ex-type. After 7 days on oatmeal agar, in 9 cm diam plates, at 25°C.

**Figure 2:**
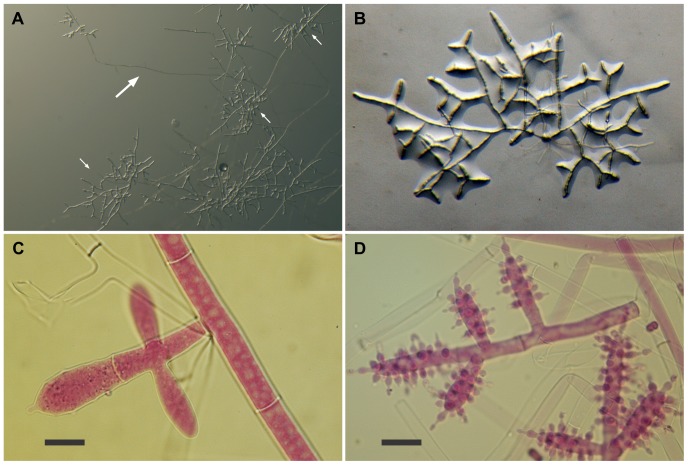
Escovopsis moelleri. (**A–B**) Growth habit, note stolons (long arrow) formed at the colony edge with rhizoids (short arrows) developing on the agar surface; (**C–D**) Details of conidiogenesis showing early development of the clavate vesicles (**C**, scale bar = 10 µm), and, a later stage covered with swollen, short-necked phialides (**D**, scale bar = 20 µm).

**Figure 3:**
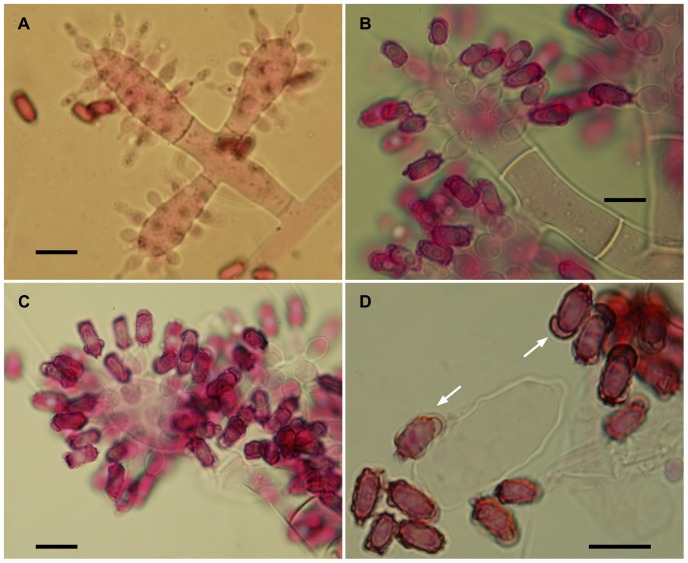
Escovopsis moelleri. (**A–D**) Older stages of vesicle development showing darkening conidia with thickened rugose walls and apical cap-like structures (arrows); note the short-lived or evanescent vesicle (**D**). All scale bars = 10 µm.

**Figure 4:**
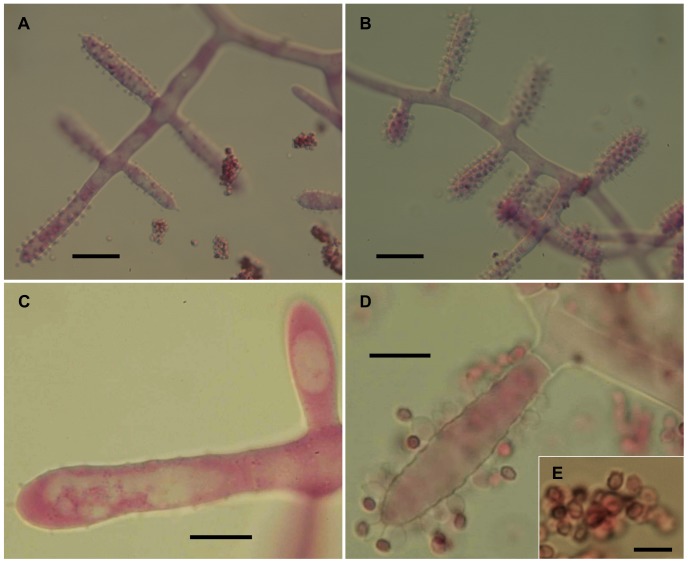
Escovopsis microspora. (**A–B**) Details of conidiogenesis with clavate vesicles and swollen, short-necked phialides producing chains of conidia (scale bar = 20 µm); (**C–D**) Older evanescent vesicles with dark spores (scale bar = 10 µm); (**E**) Inset, showing conidial ornamentation (scale bar = 5 µm).

**Figure 5:**
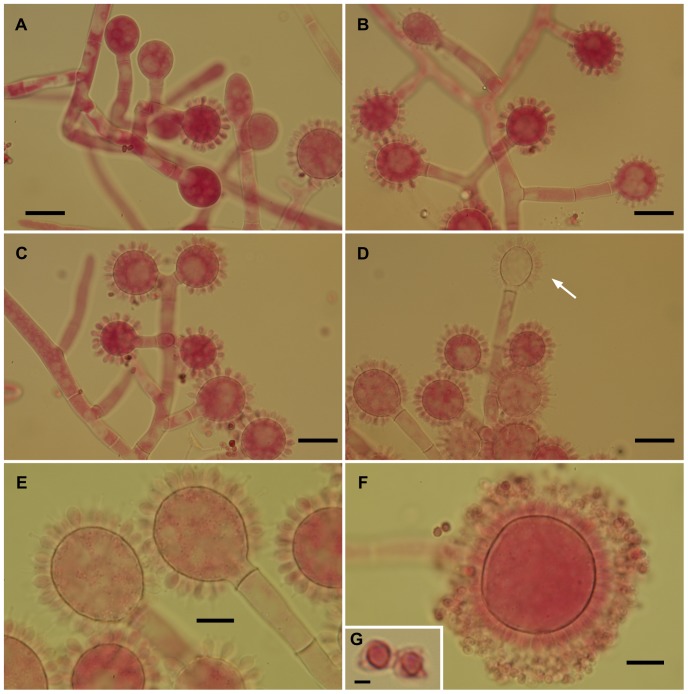
Escovopsis lentecrescens. (**A–D**) Stages of conidiogenesis, resulting in evanescent heads (**D**, arrow), all scale bars = 20 µm; (**E–F**) Paratype, faster-growing strain with more evanescent heads (scale bar = 10 µm), inset (**G**) showing detail of spore veil or coat (scale bar = 2 µm).

**Figure 6:**
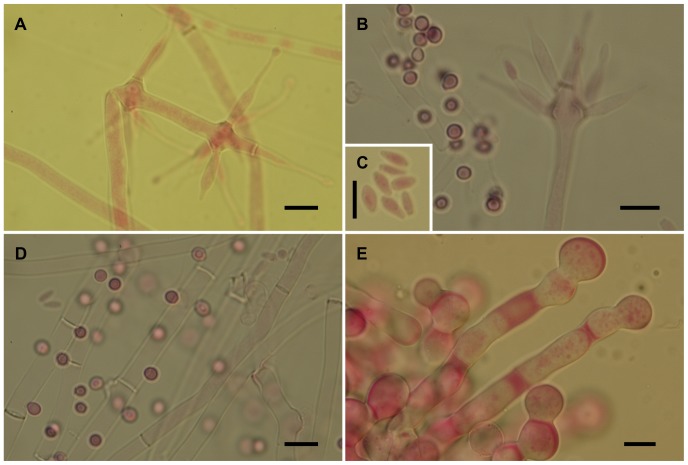
Escovopsioides nivea. (**A–B**) Conidiophores bearing both terminal and intercalary vesicles with few cylindrical, subulate phialides tapering gradually to a long neck region, and hyaline, thin-walled conidia (inset, **C**)—distinguished from the sphaerical darker aleurioconidia (**B**, left above inset); (**D**) Aleurioconidia emerging directly from hyphae; (**E**) Chlamydospores sensu lato formed in glistening white chains or ropes, densely guttulate. All scale bars = 10 µm.

**Figure 7:**
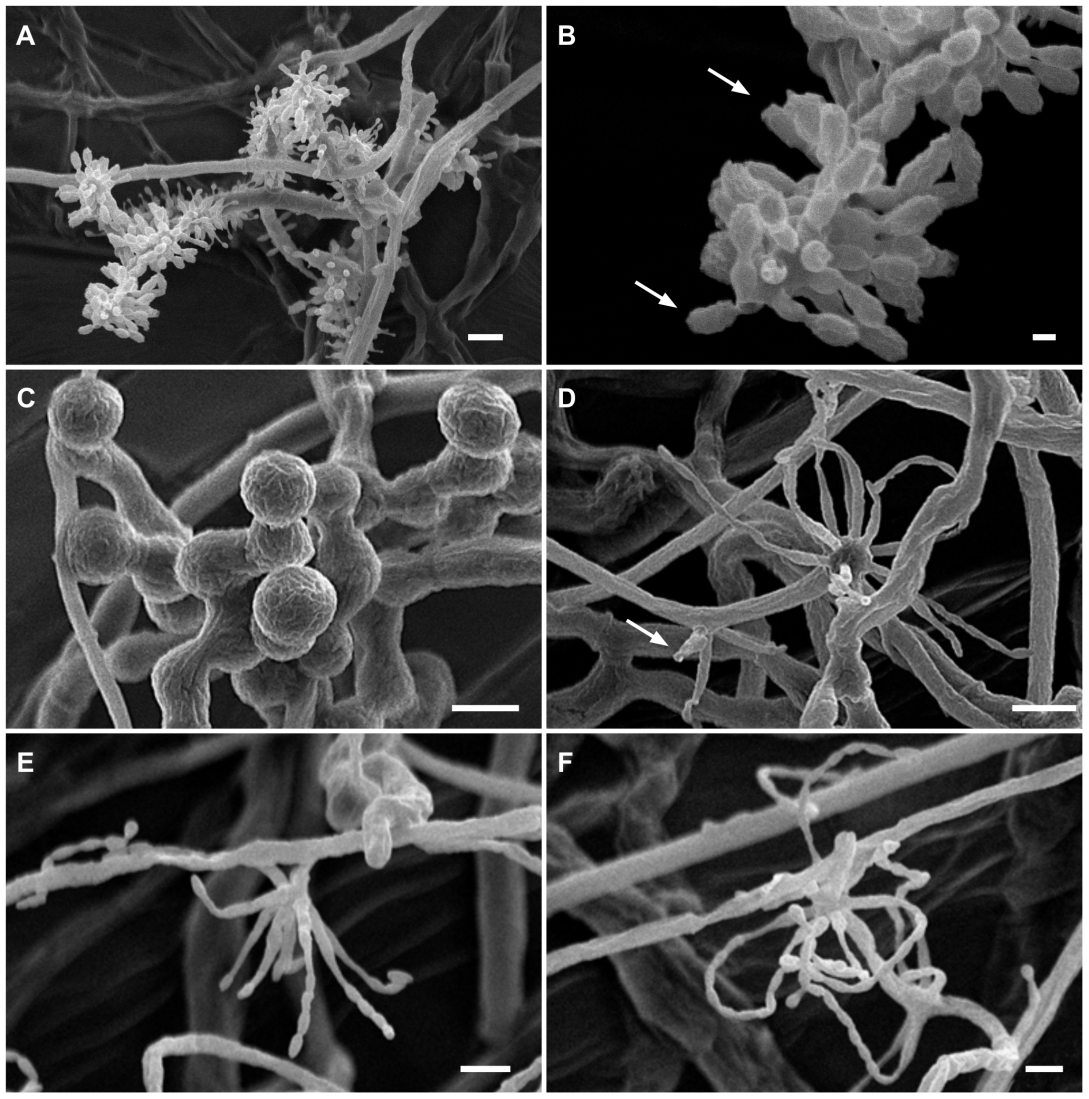
Details of conidiogenesis and spore morphology, as revealed by Critical-Point Drying SEM. (**A**) *Escovopsis moelleri*, showing branching and vesicle formation (scale bar = 10 µm); (**B**) Detail of conidial morphology, with ornamentation and apical cap (arrows) (scale bar = 2 µm); (**C**) *Escovopsioides nivea*, chains of chlamydospores sensu lato revealing cryptic surface ornamentation or mucilaginous deposit (scale bar = 10 µm); (**D**–**F**) *Escovopsioides nivea*, (**D**) showing both terminal vesicle and phialides produced laterally on slight swelling (arrow) (scale bar = 10 µm); (**E–F**) Lageniform phialides with long chains of conidia (scale bar = 5 µm).

**Figure 8:**
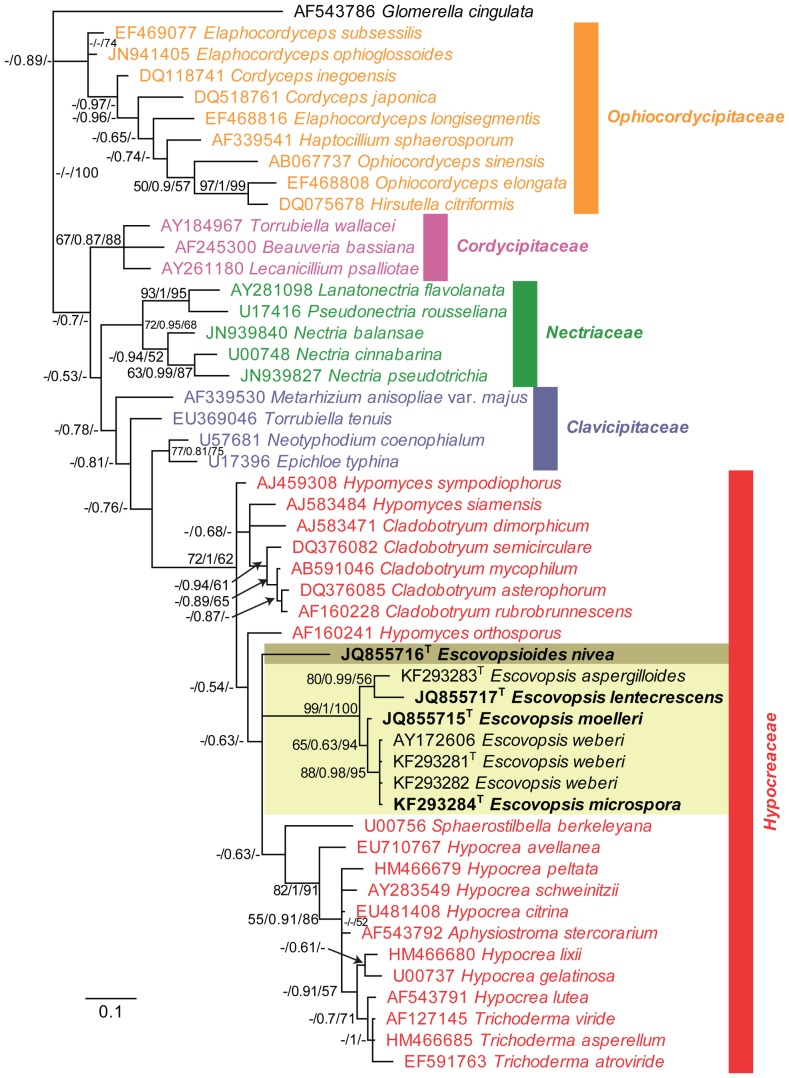
Fifty-percent majority rule tree obtained from the Bayesian analysis of LSU DNA sequence data. The *Escovopsis* and *Escovopsioides* clades are highlighted in shaded boxes. In these clades, a superscript T denotes sequences obtained from (ex-) type strains. Regular type taxa in the clade ascribe sequences obtained from GenBank, which were included for the purpose of phylogenetically placing the new species. Except for some rearrangements at the deeper nodes (see TreeBASE), this Bayesian consensus tree is topologically identical to trees obtained from maximum parsimony (MP) and maximum likelihood (ML) analyses. Numbers at tree nodes are Maximum likelihood bootstrap support values (integer value up to 100), posterior probabilities support values obtained from Bayesian analysis (fraction up to 1) and parsimony bootstrap support values (integer value up to 100), respectively. Colour taxa indicate representatives from the hypocrealean families shown on the tree. The scale bar represents the expected number of changes per site. *Glomerella cingulata* was used to root the tree.

**Figure 9:**
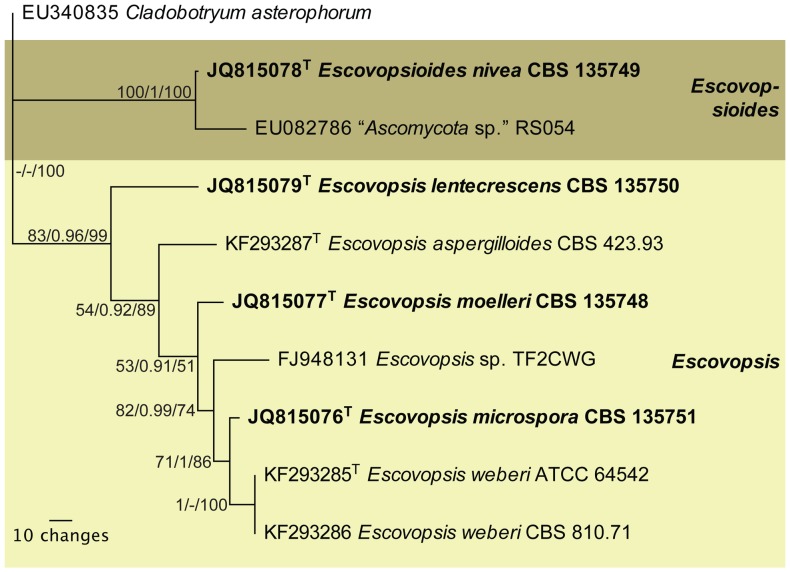
Single most parsimonious tree obtained from a parsimony analysis of ITS DNA sequence data. The *Escovopsis* and *Escovopsioides* clades are highlighted in shaded boxes. In these clades, a superscript T denotes sequences obtained from (ex-) type strains. Bold type taxa are described as new species in this manuscript. This maximum parsimony (MP) tree is topologically identical to trees obtained from Bayesian (B) and maximum likelihood (ML) analyses. Numbers at tree nodes are Maximum likelihood bootstrap support values (integer value up to 100), posterior probabilities support values obtained from Bayesian analysis (fraction up to 1) and parsimony bootstrap support values (integer value up to 100), respectively. The scale bar indicates the number of changes. Based on the LSU tree, *Cladobotryum asterophorum* was used to root the tree.

The isolates from fungal gardens and middens were grouped initially into four morphotypes, based on colony colour (Fig. 1), vesicle shape and spore morphology (Figs. 2–6; S1; 7). These characters proved to be robust and were confirmed by the results of the molecular characterization (Figs. 8; 9). Three of these morphotypes readily fall into the concept of the genus *Escovopsis* and two distinct forms can be delimited: the *E. weberi*-type with clavate-cylindrical vesicles (Figs. 2–4); the *E. aspergilloides*-type with globose vesicles (Fig. 5). These are described as new species and keyed out from each other and from the other two known species in the genus (see Table 1). However, the fourth morphotype shows morphological characters which fall outside of the *Escovopsis* concept by forming: terminal (Fig. 6B) and intercalary vesicles (Fig. 6A); pronounced lageniform (Figs. S1A; 7D, F) to subulate phialides (Figs. 6A, B); and hyaline conidia in long chains (Fig. 7F). In addition, two other spore types are produced: bead-like chains of hyaline chlamydospores sensu lato (Figs. 6E; S1D; 7C; S2B); and discrete, dark-walled aleurioconidia [40], arising directly from the vegetative mycelium (Figs. 6B, D; S1C). Separation of this morphotype at the generic level is also supported by the molecular data and a new genus is proposed to accommodate it.

**Table 1:** Key to the genus *Escovopsis* and its relatives.

1. Colonies predominantly white	Escovopsioides nivea
1. Colonies turning golden yellow to dark brown	2
2. Conidiophores vesiculate, cylindrical to clavate	3
2. Conidiophores vesiculate, predominantly aspergilloid	5
3. Conidia <5 µm in length	4
3. Conidia >8 µm in length	*Escovopsis moelleri*
4. Conidia 3–4 µm in length	*E. weberi*
4. Conidia <3 µm in length	*E. microspora*
5. Colonies slow growing (<1 mm/day)	*E. lentecrescens*
5. Colonies fast growing (>5 mm/day)	*E. aspergilloides*

### Morphological characterization

Previously, in order to validate new fungal taxa, the formal description had to be preceded by a Latin diagnosis. However, since the start of 2012, changes to the International Code of Botanical Nomenclature—now called “the International Code of Nomenclature for algae, fungi, and plants (ICN)”—have meant that this is no longer a requirement. Nevertheless, it is recommended that a diagnosis (a short statement on how the new taxon differs from related taxa), which may be in Latin or English, should still precede the main description [41]. This format is adopted here. Similarly, electronic material published online as a PDF with designated ISSN or ISBN codes will now constitute a valid publication for new names of organisms [42].

***Escovopsis moelleri*** H.C. Evans & J.O. Augustin sp. nov.

MycoBank: MB800440

GenBank: JQ815077; JQ855712–JQ855715

Etymology: Named in honour of A.F.W. Moeller who pioneered the work on fungal gardens of ants and provided the first illustrations of *Escovopsis*.

Differs from the type species, *Escovopsis weberi*, in spore morphology—conidia of *E. moelleri* are much larger and heavily ornamented—and from *E. aspergilloides* in the clavate to cylindrical vesicles, and from both by DNA sequences.

Type: BRAZIL. Minas Gerais: Viçosa, Mata do Paraíso, 700 m, 9 Mar 2010, *J.O. Augustin & H.C. Evans*, isolated from fungal garden of *Acromyrmex subterraneus molestans* Forel (holotype IMI 501176; isotype DOA626-VIC 31753, CBS 135748).

*Colonies* fast-growing, 5–6 cm diam after 7 days on PCA at 25°C, covering a 9-cm diam plate within 14 days; greyish-white becoming brown as conidia mature; but slower, more compact growth on OA (Fig. 1C); aerial mycelium dense reaching lid of plate, 8–10 µm diam; greyish-brown reverse; colony periphery uneven or feathery due to abundant, spreading stolons forming dichotomously-branching rhizoids (Figs. 2A, B): growth similar, but slightly faster on PDA. *Conidiophores* formed from aerial hyphae, cylindrical; branching orthogonal cruciform to monopodial, 55–90×10–16 µm, smooth, hyaline to subhyaline. *Vesicles* produced laterally and apically on the branches (Figs. 2C, D); mainly clavate, 20–40 (-45)×10–16 µm, also cylindrical and up to 80 µm long. *Phialides* arranged in uniseriate rows along the vesicles; ampulliform, 6–9×4–5 µm, with a short, narrow neck (2×>1 µm) (Figs. 3A, B): those on cylindrical vesicles longer and narrower, averaging 10×3 µm, tapering to a narrow neck up to 4 µm in length. *Conidia* produced in short, non-persistent, basipetal chains; initially hyaline and smooth, becoming brown, thick-walled and conspicuously warty, oblong, 7–10×3.0–3.5 µm, with a truncate base and developing a distinct cap-like structure apically (Figs. 3C, D). Other spore forms not observed.

#### Commentary

*Escovopsis weberi* produces similarly-shaped vesicles, which are more cylindrical, less variable in form and longer (43–58×11–14 µm) than *E. moelleri* [15]—although, it should be noted that subsequent descriptions and illustrations also include distinctly clavate vesicles [17], [40]—with smaller, typically globose phialides (3.0–4.5 µm diam, at base). However, the species are most easily separated on conidial size. In culture, growth of *E. weberi* was described as slow, but with no quantitative data [15]; conversely, Seifert et al. [17] reported fast growth of this species, covering a 9-cm diam plate within 5 days at 25°C on most media.

***Escovopsis microspora*** H.C. Evans & J.O. Augustin sp. nov.

MycoBank: MB800442

GenBank: JQ815076; KF293284

Etymology: With reference to the small conidia.

Distinguished from *Escovopsis moelleri* and *E. weberi* by the similarly ornamented but smaller conidia; and from both by DNA sequences.

Type: BRAZIL. Minas Gerais: Viçosa, Mata do Paraíso, 700 m, Apr 2010, *J.O. Augustin & H.C. Evans*, isolated from fungal garden of *Acromyrmex subterraneus molestans* (holotype IMI 501177; isotype DOA629-VIC 31756, CBS 135751).

*Colonies* fast-growing, on PCA and OA attaining 4.5–5.5 cm diam after 7 days at 25°C, relatively low with sparse aerial mycelium; khaki brown centrally with a distinct snow-white periphery (Fig. 1E), reaching sides of a 9-cm diam plate after 14 days and becoming uniformly brown with abundant dark brown exudate droplets; whitish-brown reverse; stolons and rhizoids absent, colony edge relatively even; on MA producing a deep reddish- brown diffusate. *Conidiophores* formed from aerial mycelium, cylindrical; branching orthogonal, opposed-cruciform to monopodial; up to 200 µm long, 6–8 µm wide, forming vesicles laterally and apically (Fig. 4A). *Vesicles* predominantly cylindrical, 45–60×7–8 µm (Figs. 4A, B), occasionally clavate, (20-) 28–40×8–13 µm (Fig. 4C), covered with a layer of phialides. *Phialides* globose at base, 3 µm diam, with narrow (>1 µm diam) needle-like neck, 1–3 µm in length (Fig. 4D). *Conidia* produced in persistent, basipetal chains, subhyaline becoming thick-walled, brown and warty (Fig. 4E): globose, averaging 2–3 µm diam, to ovoid, 2.5×1.5 µm, with a truncate base. *Chlamydospores* formed occasionally in culture, especially on MA; hyaline, globose, 15–20 µm, rarely sub-globose, as terminal swellings of dichotomously-branching hyphae; coalescing into plates or sheets on the agar surface.

#### Additional specimen examined

BRAZIL. Minas Gerais: Viçosa, Mata do Paraíso, Jan 2011, *H.C. Evans*, growing over and isolated from midden of *Acromyrmex subterraneus subterraneus* Forel (paratype IMI 501178) (Fig. S3D).

#### Commentary

**Figure 10:**
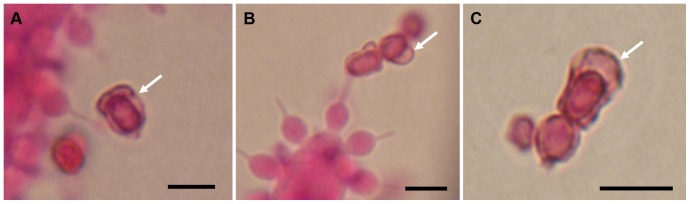
Light microscopy of *Escovopsis* ex type cultures. (A–B) *Escovopsis weberi* ex type culture, ATCC 64542: Spore chain still attached to phialide on cylindrical vesicle, showing the relatively early development of the pigmented outer spore wall or sheath (**A**); Close-up of another conidium with prominent outer sheath (**B**). Most conidia are older and detached before the pigmentation and separation of the outer wall or sheath become obvious. (**C**) ***Escovopsis aspergilloides*** **ex type culture, CBS 423.93:** Detached spore chain in various stages of maturation, showing pigmentation and prominent outer walls. Arrows indicate the outer caps. All scale bars = 5 µm.

In addition to conidial size, cultural characters can also be used to separate this species from *E. moelleri*, such as the absence of rhizoids, presence of brown exudate and the distinctive white and even periphery around the colony edge of *E. microspora* (Fig. 1E). Separation from *E. weberi* is more problematic, however, since conidial sizes overlap; conidia of the former being described as “globose to ovoid, hyaline, smooth, 2.2–3.3×2–3 µm” [15]. Nevertheless, an examination of the type of *E. weberi* (ATCC 64542) revealed that this description is erroneous, being based on immature spores, and that spore wall pigmentation and ornamentation develop later: a characteristic shared by all species of the genus due to the development of a distinct outer wall or mucilaginous sheath (Figs. 3D; 4E; 5G; 10A–C).

***Escovopsis lentecrescens*** H.C. Evans & J.O. Augustin sp. nov.

MycoBank: MB800441

GenBank: JQ815079; JQ855714–JQ855717

Etymology: Based on the markedly slow growth rate in vitro compared to all other described species.

Species similar to *Escovopsis aspergilloides* in possessing globose vesicles: readily separated by cultural characters, especially the slow colony growth, and in micro-morphology by the larger, heavily-ornamented conidia, and by the DNA sequences.

Type: BRAZIL. Minas Gerais: Viçosa, Mata do Paraíso, 700 m, Apr 2010, *J.O. Augustin & H.C. Evans*, isolated from fungal garden of *Acromyrmex subterraneus subterraneus* (holotype IMI 501179; isotype DOA628-VIC 31755, CBS 135750).

*Colonies* on PCA and OA very slow growing, no recordable growth after 7 days (Fig. 1D); reaching 0.6–0.7 cm diam after 14 days and 1.5–1.7 cm after 21 days at 25°C; pinkish brown centrally with a white, even periphery of immature conidiogenous structures: *mycelium* sparse and colonies consisting of conidiophores arising directly from the agar or from aerial hyphae; growth equally poor on PDA and MA. *Conidiophores* cylindrical; branching orthogonal, opposed-cruciform to monopodial; hyaline, up to 200–300 µm in length and 9–10 µm diam. *Vesicles* produced laterally and terminally on short side branches, 30–40×6–8 µm, consistently 2–3 septate; variable in form, mainly globose and aspergilloid, (16-) 18–28 µm diam (Fig. 5), occasionally clavate, 23–29×20–23 µm. *Phialides* subglobose, 3.5–5.0×2.5–3.5 (-4.0) µm, with a short, abrupt, spike-like neck; aberrant structures also produced on some vesicles, extending to form ampulliform to cylindrical phialides with long tapering necks. *Conidia* in basipetal chains, ovoid to subglobose, 3.0–4.0×2.0–2.5 µm; becoming brown and echinulate to verruculose, older conidia with a loose, dark outer covering or veil (Fig. 5G).

#### Additional specimen examined

BRAZIL. Minas Gerais: Viçosa, Mata do Paraíso, Jan 2011, *H.C. Evans*, associated with and isolated from a midden of *Acromyrmex subterraneus subterraneus* (paratype IMI 501180).

#### Commentary

The paratype is slightly faster growing, 1.1–1.3 cm diam after 14 days; occasionally producing intercalary chlamydospores, globose (10–15 µm) to subglobose (15×10 µm). This species is also common on the middens of *A. subterraneus subterraneus*. The conidia of the new species are slightly larger than *E. aspergilloides* (2.5–3.7×1.5–2.0 µm). However, as with *E. weberi*, there are discrepancies concerning conidial descriptions; in the original diagnosis of *E. aspergilloides* [17], for example, there is no mention of the surface or colour of the conidia, although from the illustration they appear to be smooth and non-pigmented. Moeller [19] had described them as becoming brown with age. Once again, the defining characteristics of *Escovopsis* spores–pigmentation and ornamentation–had been overlooked since these were found when the type of *E. aspergilloides* (CBS 423.93) was examined in this study.

***Escovopsioides*** H.C. Evans & J.O. Augustin gen. nov.

MycoBank: MB800474

Etymology: In reference to similarities with the genus *Escovopsis* in producing a brush-like anamorph with phialides on well-defined vesicles.

Differs from *Escovopsis* by: the absence of pigmentation; the lageniform phialides produced on terminal and intercalary, globose vesicles; the hyaline, smooth conidia in long chains, and the DNA sequences.

Type species: ***Escovopsioides nivea*** H.C. Evans & J.O. Augustin sp. nov.

MycoBank: MB800475

GenBank: JQ815078; JQ855713–JQ855716

Etymology: In reference to the characteristic snow-white colonies both in culture and on the fungal gardens and middens.

Type: BRAZIL. Minas Gerais: Viçosa, Mata do Paraíso, 700 m, Jan 2011, *H.C. Evans*, growing over and isolated from midden of *Acromyrmex subterraneus subterraneus* (holotype IMI 501181; isotype DOA627-VIC 31754, CBS 135749).

*Colonies* on PCA and OA growing rapidly, up to 6–7 cm diam after 7 days at 25°C and reaching the edge of a 9-cm diam plate within 10 days; white, cotton-like aerial mycelium with abundant production of chains of chlamydospores s. l., uniting into snow-white, silvery ropes or strands (Fig. 1F); white reverse: older colonies becoming creamish. Similar growth on PDA and MA, reaching 3.5–4 cm diam after 14 days; stolons with dichotomously-branching rhizoids formed around periphery, giving it an uneven appearance. *Chlamydospores sensu lato* hyaline, smooth- and thin-walled, globose, 15–18 µm diam, densely guttulate, produced laterally from swollen hyphae in monilioid chains or clusters (Figs. 6E; S1D; 7C). *Aleurioconidia* produced as lateral swellings from the walls of the aerial mycelium, abundant; sub-hyaline, sphaerical, 3–4 µm diam, smooth- and thick-walled (Fig. 6D). *Conidiophores* arising from aerial mycelium: hyaline, smooth-walled, multiseptate, cylindrical, 40–70 (up to 150) µm in length, 5 µm diam at base narrowing towards apex before swelling into a globose vesicle, 8–10 µm diam, often proliferating terminally to produce several intercalary vesicles in succession (Fig. 6A). *Phialides* formed in small groups (5–10) on vesicles, but also singly on discrete lateral swellings (Fig. S1A); septate at base, sometimes proliferating terminally and laterally (polyphialidic); variable in shape and size: predominantly lageniform, 18–35×2.5–3.5 µm, tapering gradually to a blunt or long thin neck, 1 µm diam (Figs. 6A, B; S1A–C); occasionally ampulliform, 10–20×2–3 µm, with a short neck. *Conidia* in long, persistent chains; hyaline, smooth- and thin-walled, limoniform to clavate, (3-) 5–8×(1.5-) 2–3 µm, ending abruptly in or tapering to a truncate base (Fig. 6C).

#### Additional specimen examined

BRAZIL. Minas Gerais: Viçosa, Mata do Paraíso, Apr 2010, *J.O. Augustin & H.C. Evans*, isolated from fungal garden of *Acromyrmex subterraneus subterraneus* (paratype IMI 501182).

#### Commentary

As mentioned previously, the first mycologist to observe this fungus was Moeller in 1893 and his monograph presents accurate drawings of the new genus, including all three spore types [19]. Initially, the paratype was distinguished as a separate morphotype since it produced only aleurioconidia and chlamydospores s. l. Subsequently, sporogenesis was found to be a highly variable character, as phialides and conidia developed intermittently in subcultures. The type species of this new genus has relatively few morphological features in common with species of *Escovopsis*, apart from producing phialides on vesicles. Phialide form and conidial morphology, especially the long chains (see Figs. 7D, F), share more similarities with species in the genus *Paecilomyces*, most notably, *P. variotii* Bain. However, the combination of lageniform phialides on terminal and intercalary vesicles makes *Escovopsioides nivea* unique. Indeed, the species can readily be identified in illustrations provided in a dissertation on the fungi associated with nests of leaf-cutting ants in Brazil [43], where it is listed as *Moniliella suaveolens* (Lindner ex Lindner) Arx. We refer to the swollen spores produced in monilioid chains as chlamydospores s. l., because these are not thick-walled perennating cells produced endogenously [3], and their function is obscure. Kreisel [16] refers to similar structures found in his *Escovopsis* ( = *Phialocladus*) isolate from Cuba, as “makroconidien”, which were not found in the supposed “cospecific” type from Brazil [15]. For the present, we use the term chlamydospore sensu lato until their role in the life cycle is better understood.

### Molecular characterization and phylogenetic analyses

We obtained partial sequences for two non-protein coding genes of the nuclear ribosomal RNA gene operon, namely partial **LSU** (Fig. 8; 504 bp including alignment gaps, conserved sites (C) = 356, variable sites (V) = 34, parsimony informative sites (Pi) = 114, limited to 1000 equally most parsimonious trees), and **ITS** (Fig. 9; 399 bp including alignment gaps, conserved sites (C) = 268, variable sites (V) = 60, parsimony informative sites (Pi) = 71, single most parsimonious tree found); as well as part of the protein coding gene, translation elongation factor 1-alpha (**EF1-α** Fig. S4: 716 bp including alignment gaps, conserved sites (C) = 340, variable sites (V) = 62, parsimony informative sites (Pi) = 314, limited to 1000 equally most parsimonious trees; Fig. S5: 373 bp including alignment gaps, conserved sites (C) = 172, variable sites (V) = 28, parsimony informative sites (Pi) = 173, limited to 1000 equally most parsimonious trees). Phylogenetic analyses using Maximum Parsimony (MP), Maximum Likelihood (ML) and Bayesian methods on the ITS and LSU datasets all resulted in reconstructed trees with similar topology (available in TreeBASE, www.treebase.org). The LSU tree (Fig. 8) places the *Escovopsis* species in a well-supported clade in the *Hypocreaceae* (*Hypocreales*) with the novel genus, represented by *Escovopsioides nivea*, as a distantly related sister genus. *Escovopsis* and *Escovopsioides* are included in the *Hypocreaceae* with a posterior probability (PP) value of 1 and the parsimony bootstrap (PB) value of this *Hypocreaceae* clade was 62%. However, the sister relationship amongst *Escovopsioides* and the *Escovopsis*-clade and “*Hypocrea*/*Trichoderma*”-clade has a PP value of only 0.63 and this association is not resolved in the parsimony bootstrap. Nevertheless, the *Escovopsis* clade itself (excluding *Escovopsioides*) is supported by PP = 1 and PB = 100%. For LSU, the Bayesian trees identified—with high posterior probability support—two sub-groups within the *Escovopsis* clade made up of *Escovopsis weberi*-like isolates, comprising *E. weberi* and *E. microspora*; and, *Escovopsis aspergilloides*-like isolates, comprising *E. aspergilloides* and *E. lentecrescens*; and, finally, placement of *E. moelleri* intermediate between these two groups. A similar association between *E. weberi* and *E. microspora* was observed in the ITS phylogeny (Fig. 9); however, the remaining three species represented separate lineages in the well-supported *Escovopsis* clade. Also, in the ITS phylogeny (Fig. 9), *Escovopsioides nivea* formed a distinct lineage, clustering with a sequence listed in GenBank as “*Ascomycota* sp.” (isolated from an *Acromyrmex lundii* (Guérin-Méneville) nest in Brazil) but which in fact could represent an additional species of *Escovopsioides*. Megablast searches of NCBI's GenBank nucleotide database using the obtained EF1-α sequences yielded no high identity hits with other sequences in GenBank but did reveal significant similarity (up to 97% identity) with other *Escovopsis* sequences or species of *Hypocreales*, confirming the affinity of our sequences with related sequences in GenBank. However, preliminary phylogenetic analyses using two different lengths of these sequences (Figs. S4, S5) did not result in robust phylogenies as this part of the EF1-α gene does not appear to be informative enough at the species—and even genus—level in the current study, in order to build a well-resolved phylogenetic tree that includes several species from different families of the *Hypocreales*. In both the phylogenies derived from the longer (Fig. S4) and shorter (Fig. S5) sequences, several of the families are not monophyletic (see colours in these two figures), irrespective of the algorithm used, and also several sequences labeled as *Escovopsis* are interspersed between these families. However, most of the *Escovopsis* sequences do cluster together to some degree. Of some interest is the basal position of our new genus in the second *Hypocreaceae* lineage (Fig. S5), which could imply that some of the “*Escovopsis*” lineages that are located between the two *Hypocreaceae* lineages might represent one or more additional novel genera. It is a pity that more ITS and/or 28S nrDNA sequences are not available for these basal lineages of *Escovopsis* in the EF1-α phylogenies as it remains an open question whether these might actually represent additional *Escovopsis*-like genera or whether it is a lack of resolution for the region of EF1-α used in our and previous studies [22], [28], [33]. A manual inspection of the alignment indicates that the part of EF1-α included in the current study does not contain any introns that could have resulted in a more robust phylogeny and that many of the polymorphisms appear to be homoplastic. This is the reason why this work presents the phylogeny of *Escovopsis* and its relatives based predominantly on ITS and LSU. Finally, the megablast search did confirm that no other identical EF1-α sequences are present in GenBank at this time.

#### Commentary

The molecular results corroborate the morphological studies enabling three new species to be added to the genus *Escovopsis*, as well as the recognition of the new genus *Escovopsioides*. The phylogenetic analyses of ITS and LSU demonstrate the monophyly of the genus *Escovopsis*, in agreement with other phylogenetic studies on *Escovopsis* [22], [33], and also allows for the erection of a new genus, with good phylogenetic support.

### Fungal sampling

*Escovopsis*-type infestation of *Acromyrmex* colonies is common, as shown by the survey results (Table 2). Fungal colonies emerged from the garden pieces within two days. Moreover, multiple *Escovopsis*-type species were isolated from single fungal gardens (Table S2), as well as from individual fungal garden pieces on agar samples (data not shown).

**Table 2:** Results of sampling of *Escovopsis*-like species isolated from fungal gardens of *Acromyrmex* spp. in Viçosa, Minas Gerais, Brazil.

Fungal species	*Acromyrmex niger*		*Acromyrmex subterraneus molestans*		*Acromyrmex subterraneus subterraneus*		% of nests infected (n = 25)
	N = 2; P = 100%		N = 5; P = 45%		N = 8; P = 66%		
	Top	Bottom	Top	Bottom	Top	Bottom	
	n = 2	n = 2	n = 11	n = 11	n = 12	n = 12	
*Escovopsis moelleri*	–	–	1 (MB)	2 (MB) 2 (MP)	–	1 (MB)	24
*Escovopsioides nivea*	1 (MP)	1 (MB) 1 (MP)	1 (MB) 1 (MP)	2 (MB) 2 (Rep)	3 (RC) 1 (Rep) 1 (MP)	1 (MB) 3 (MP)	72
*Escovopsis microspora*	–	1 (MP)	1 (MB) 2 (Rep)	2 (MB) 2 (Rep) 2 (MP)	–	1 (Rep) 1 (MP)	48
*Escovopsis lentecrescens*	–	–	–	–	–	1 (MB) 1 (MP)	8

*n* = Number of sub-colonies sampled; N = Number of sub-colonies infected; P = Proportion of infected sub-colonies.

MB = Mata da Biologia (UFV campus); Rep = Represa UFV (UFV campus); MP = Mata do Paraíso.

Infection of fungal gardens by *E. moelleri* and *E. microspora* was frequent (24% and 48% of 25 sub-colonies, respectively) and consistent with previous reported patterns of high incidence of *Escovopsis* among *Acromyrmex* ants [13], [22], [24], [28]. However, *E. lentecrescens* was isolated in only 8% of the 25 samples. This may be due to its slow-growing habit, enabling faster-growing species to outcompete *E. lentecrescens* in the isolation plates. Conversely, the incidence of *Escovopsioides nivea* was high, and reached 72% of the 25 sub-colonies sampled.

Previous work has shown that infection rates of *Escovopsis* among the leaf-cutting ants from Central America can vary between 42.9–51.4%, out of 49 and 29 colonies of *Atta* and *Acromyrmex*, respectively [13]. A survey in southern Brazil revealed that 27% of 37 colonies from ten different *Acromyrmex* species were positively sampled for *Escovopsis* [30], while *E. weberi* was found in 42% of 20 sub-colonies of *A. sexdens rubropilosa* Forel from south-east Brazil [44]. Moreover, a range of fast-growing fungal species—in particular, those belonging to the genera *Syncephalastrum* J. Schröt. and *Trichoderma* Pers.—came out from single garden pieces (data not shown). This means that ‘stronger’ fungal isolates probably outcompeted ‘weaker’ ones with the methodology used, which may have underestimated fungal diversity, as well as the incidence of species such as the slow-growing *E. lentecrescens*, for example. This species may be a more prevalent symbiont in the in vivo system—perhaps using other competitive strategies, such as antibiosis—but a poor competitor in vitro. It also suggests that a more intricate coevolutionary history exists between *E. lentecrescens* and *Leucoagaricus*, so that the parasite is nutritionally more dependent on its fungal host. Alternatively, fast-growing species, such as *Escovopsioides nivea*, could have been favored by the sampling method used in this study, essentially, based on a single, nutrient-rich medium. Potentially, therefore, the use of selective media could reveal an even higher diversity of *Escovopsis*-like mycoparasites.

It has previously been shown that individual colonies of leaf-cutting ants from Central America harbor genetically different *Escovopsis* strains and in vitro bioassays showed no apparent antagonistic interaction between them [32]. The same trend was observed in the present study. The *Escovopsis* and *Escovopsioides* species isolated from individual *Acromyrmex* colonies in our study also did not appear to inhibit each other, and we observed that up to three different *Escovopsis* species emerged from individual garden pieces on agar [J.O. Augustin, pers. obs.]. In addition, the four new species described here showed no apparent antagonistic interactions in dual cultures [J.O. Augustin, unpubl. data.].

### Linking morphology to ecology

#### Sporogenesis

Genetic studies have demonstrated the long evolutionary history of *Escovopsis* and attine ants [22], [27], [28] and, at a broader level, the morphology of each new species described here also reflects parasite adaptation to its fungal host, particularly based on the form and function of the vesicles and conidia.

The two sub-clades differ in vesicle shape—aspergilloid or clavate/cylindrical—but the relative merits of these shapes, in terms of increasing spore production, is difficult to assess. Of interest is the evanescent or ephemeral nature of the vesicles and phialides: designed for production of spore inoculum over an extremely short period; perhaps, in order to maximize resources or to exploit narrow windows of opportunity for dispersal. However, there can be no doubt about the form and function of the conidia; showing adaptations for dispersal, dormancy and adherence. Even without the aid of electron microscopy, the conidia of all three species can be seen to develop outer walls with varying degrees of complexity, especially in wall ornamentation. In the relatively large-spored *E. moelleri*, for example, the mature conidia become heavily pigmented with distinctly rugose walls and a well-defined apical cap (Fig. 3D). The other two species, with significantly smaller spores, show permutations of these features (Figs. 4E; 5G). Thus, the conidia of all three species appear to be adapted for attachment to an arthropod vector. Alternatively, the different outer walls exhibited by *Escovopsis* conidia would help the mycoparasite to “adhere” to the fungal garden, forcing the ants to perform alternative strategies of hygiene to remove spores away from gardens. Supporting this view are the reported long-term infections of fungal gardens by this parasite [13], [24], the intricate prophylactic and hygienic behavioral repertoire performed repeatedly by founding queens [45]–[48] and workers in every aspect of colony maintenance [19],[36],[49],[50]; as well as protective symbiotic associations with microorganisms [35], [37], [51]–[55].

In addition, spore melanization and wall thickening are traits associated with long-term survival. Indeed, replicating cultures of these species, using older conidia as the sole inoculum, can frequently result in no colony establishment, indicating the involvement of a dormancy mechanism. Empirical experiments are now showing that this dormancy can be broken by the presence of the fungal symbiont, *Leucoagaricus*, in the culture medium [Augustin et al., in prep.]. There are no suggestions from the original descriptions of either *E. weberi* or *E. aspergilloides* [15], [17] that the conidia have similar ornamentation. Nevertheless, from the SEM micrographs presented [15], [17], there are indications that the conidia may have a cryptic mucilaginous covering or subtle ornamentation. This has now been confirmed from our examination of the type cultures of these two species, as described previously. In our opinion, amended descriptions of the genus *Escovopsis*, as well as the two designated species, are now warranted since the phoretic nature of the mature conidia is considered to be a critical generic trait ignored in previous studies.

***Escovopsis***: Anamorphic *Hypocreales*; *conidiophores* hyaline, branched, vesiculate; *vesicles* cylindrical to globose, evanescent; *phialides* hyaline, swollen at base, extending to a narrow neck; *conidia* in short basipetal chains, aseptate, hyaline at first becoming pigmented with an ornamented or mucilaginous brown outer coat or sheath, phoretic.

Note: In the original description of the genus [15], there is no mention of spore morphology; whilst the latest generic diagnosis [40] describes the spores as hyaline, dry ameroconidia.

***Escovopsis weberi***: *Vesicles* cylindrical, (20-) 24–45 (-50)×8–16 (-18) µm; *conidia* ovoid to ellipsoidal, 3.5–4.5×2.5–3.5 µm, darkening with age due to a densely pigmented outer wall or sheath (Figs. 10A, B).

Note: This re-description is based on the type culture deposited in the American Type Culture Collection (ATCC 64542), said to be isolated from “Carpenter ant fungal mass”, although this differs from the original publication which states that the fungus was isolated “during biosociological studies of *Atta* spp.”[15]. In the latter study, cultures of *E. weberi* are described as slow-growing, white at first and becoming reddish-brown with age. This is due to the pigmented spore walls which appear grey to reddish brown en masse. In the original species diagnosis, however, the conidia are described as hyaline, smooth and ovoid (2.2–3.3×2–3 µm), differing from the above; whilst the vesicles are longer and slightly narrower (43–58×11.5–14 µm) than those reported here. Moreover, the growth rate cannot be described as slow (see Fig. 1A)—*E. microspora* with a similar colony form grows faster (Fig. 1E)—but not as fast as that reported by Seifert et al. [17] who noted that an isolate of *E. weberi* (CBS 810.71) covered a 9-cm plate within 5 days at 25°C.

***Escovopsis aspergilloides***: *Conidia* ellipsoidal, 2.5–3.5×1.5–2 µm, becoming pale to tan brown with an outer pigmented wall or sheath (Fig. 10C).

Note: The above is based on an examination of the type culture (CBS 423.93) as described by Seifert et al. [17], who make no comment on the colour or composition of the spore wall, although their illustrations appear to show hyaline, smooth-walled conidia, as the subsequent generic diagnosis reflects [40].

Conversely, *Escovopsioides nivea*—characterized by its non-pigmented conidiophores and vesicles, producing relatively few phialides and hyaline, thin-walled spores in long chains—has no obvious phoretic or dormancy traits. The role of the phialoconidia, therefore, in the fungal life-cycle remains obscure. However, could the functions of horizontal dispersal and dormancy have been transferred to the other two spore forms? Intriguingly, most of the fungal resources appear to be channeled into producing the snow-white ropes or chains of chlamydospores s. l. over the fungal gardens in decline (Fig. S2A), as well as in vitro (not shown). Indeed, the paratype strain (IMI 501182) produces the chlamydospore s. l. and aleurioconidial forms in abundance, but the phialiform stage only tardily. The chlamydospores s. l. resemble a smaller version of the specialized ‘food sacs’ (gongylidia) produced by the mutualistic *Leucoagaricus* fungus (Figs. S2B–D). In addition, the production of a second conidial type—the darkened but smooth-walled (non-phoretic) aleurioconidia—must be linked to dispersal and/or dormancy.

#### Horizontal transmission

There has been a lack of evidence of how *Escovopsis* reaches its *Leucoagaricus* host within the ant nest. The few studies that have investigated this aspect indicate that *Escovopsis* does not rely on vertical transmission, since reproductive *Atta* queens, and the incipient mutualistic fungal inocula they carry in their infrabuccal pockets, have been found to be free of *Escovopsis* [13], [14]. Here, we report preliminary evidence on how horizontal transmission of *Escovopsis* could occur between *Acromyrmex* and *Atta* nests. *Acromyrmex* middens, or the waste piles from fungal gardens, along a forest trail in one of the designated study areas (see Materials and Methods), were found to be colonized consistently by at least three *Escovopsis* morphotypes: thus, the fungus has an ‘escape mechanism’ whereby it can sporulate outside of the nest.

In fact, higher *Attini* ants perform a series of task-partitioning that is crucial for colony health and maintenance. Midden building in *Acromyrmex lobicornis* Emery, for example, has been described as part of such a behavioral repertoire [56], as well as in other leaf-cutting ants [57]–[59]. However, the occurrence of ‘infected’ middens in close proximity to the nest entrance (Fig. S3D) must present a ‘health and safety hazard’ for this and neighboring colonies.

In sharp contrast, most species of *Atta*, apart from *Atta colombica* Guérin, exhibit an advanced form of nest hygiene and seal their fungal-garden waste within chambers inside the nest, and not in external middens. The occurrence of sporulating *Escovopsis* has been documented previously on the external midden of a mature *Atta colombica* colony in Panama [58], followed by colony emigration. Also from Panama, *Escovopsis* has been isolated from 70% of midden samples from 23 *A. colombica* colonies in the field [57]. Thus, in most species of *Atta*, any mycoparasites—such as *Escovopsis*—would effectively be taken out of the system. However, contamination of *Atta* foragers—either from aerial borne spores or by direct contact with phoretic spores on the forest floor, dispersed from the middens by wind, rain splash or run-off—must be an ever-present hazard. The preliminary results reported here, provide confirmation that *Escovopsis* sporulates consistently on the external fungal garden waste deposited by *Acromyrmex* workers around the nest entrances and offer the first evidence that these middens are an important source of *Escovopsis* inoculum. This, together with the dormancy and phoretic traits of the conidia, suggest a potent mechanism for horizontal transmission. Data on midden phenology will be presented elsewhere [Augustin et al., in prep.].

#### Sexual Reproduction

Despite searches in and around abandoned or moribund *Acromyrmex* nests, no traces of a sexual stage (teleomorph) have been found. It is possible that the teleomorph has been lost from the life-cycle, once the fungi entered and adapted to the attine ant symbiosis. However, this is considered unlikely given that genetic variation helps parasites keep up with their hosts over evolutionary time scales [60], [61]. Previous phylogenetic studies, as well as the work presented here, place species and strains of *Escovopsis* within the *Hypocreales* (*Ascomycota*) [22]: an order that includes not only saprobes and endophytes but also numerous animal, fungal and plant parasites [62], [63]. Remarkably, a distinctive feature of many hypocrealean fungi is their obligate parasitic evolutionary history which has been shown to be characterized by a shift in nutritional mode from plant-based (*Bionectriaceae* and *Nectriaceae*) to animal and fungal-based (*Clavicipitaceae* sensu lato and *Hypocreaceae*) [62], [63], for multiple lineages. The phylogeny presented in this study shows that *Escovopsis* lies within the *Hypocreaceae* and is closely related to mycoparasites such as *Hypomyces*, *Hypocrea* and *Trichoderma* (Fig. 8). It is plausible, therefore, to hypothesize that the ancestral *Escovopsis* may have been either a mycoparasite or a plant endophyte—and we have some evidence of endophytic ability in the species described here (Fig. S2C)—and this is supported by recent findings which show that there is a separate endophytic clade of avirulent plant symbionts within the hypocrealean, fungicolous genus *Trichoderma* [64], [65]. If so, then this window of opportunity for host shifting occurred together with the origin of ant agriculture, approximately 50 million years ago: a period of global warming which accelerated evolutionary events in the Neotropics [31]. Perhaps, therefore, we should be looking at plant substrates for the proto-teleomorph of *Escovopsis*. Nevertheless, loci such as 28S nrDNA, currently used to study the generic and family relationships in the *Hypocreales*, do not appear to provide a very high resolution or highly supported backbone to the phylogeny, making it more difficult to draw final conclusions with regard to reconstruction of the evolutionary history. A more robust multigene phylogeny for the genera and families in the *Hypocreales* and closely related orders is required to further address this issue.

## Materials and Methods

### Ethics statement

No specific permits were required for the field studies which were undertaken on property belonging to the Universidade Federal de Viçosa (UFV). No endangered or protected species were involved in the studies. No permits were required for the described study, which complied with all relevant regulations. The subject of the photograph included in Fig. S3A has given written informed consent, as outlined in the PLOS consent form, to publication of its photograph.

### Fungal sampling

Attine sub-colonies consist of the symbiont fungal garden and worker ants, different from whole colonies, in which one or more functional queens are invariably found. We collected 25 sub-colonies (ca. 200 mL of fungal garden) from three species of leaf-cutting ants in the genus *Acromyrmex: A. subterraneus molestans* Santschi, *A. subterraneus subterraneus* Forel and *A. niger* F. Smith, between March and May 2010 at sites in the hosts' sympatric range in the Atlantic rainforest of Minas Gerais (Zona da Mata Mineira): all located in the municipality of Viçosa, within the campus of the Universidade Federal de Viçosa or in a nearby forest reserve (Mata do Paraíso), 20°44′31.71″S–42°52′43.83″W, 650–700 m a.s.l., belonging to the university. *Acromyrmex* nests were chosen because they are abundant and easily accessible in the study area, being located relatively close to the soil surface (Figs. S3A–C), or buried beneath leaf litter in the case of *A. subterraneus molestans*. Samples were taken from both the top of the fungal garden, where fresh vegetation is constantly incorporated, as well as from the oldest part, at the base. Eight garden pieces (∼5 mm^3^) from each sub-colony were transferred to plates containing potato dextrose agar (PDA) [66] with antibiotics (50 mg/L of chloramphenicol) and incubated at 25°C. If *Escovopsis* emerged from a garden piece, which typically occurred within 4 days of initial isolation, the colony was scored as infected. *Escovopsis* mycelium was then subcultured on potato carrot agar (PCA) [66], for hyphal-tip isolation for molecular characterization. In addition, fungal garden waste—deposited in heaps or middens, around the nests—was sampled whenever *Escovopsis*-like overgrowths were observed (Fig. S3D).

### Morphological data

Colony morphology and growth rates of isolates were compared on oatmeal agar (OA), PDA,PCA, as well as on malt extract agar (MA) [66], at 25°C, in the dark or 12-hour light/12-hour dark. For microscopic analysis, material was mounted in lacto-fuchsin and examined with an Olympus RX51 light microscope with MicroPublisher 3.3 RTV Q imaging camera. For Scanning Electron Microscopy (SEM), fungal samples were fixed using osmium tetroxide for 24 h, then dried in a Critical Point Dryer (CPD 020, Balzers, Liechtenstein), mounted on stubs and sputter coated with gold (FDU010, Balzers, Liechtenstein). SEM images were taken with a LEO 1430 VP (Carl Zeiss, Cambridge, UK).

Specimens were deposited in the IMI Culture Collection (Centre for Agriculture and Biosciences International, Egham, Surrey, UK), as well as in the CBS-KNAW Fungal Biodiversity Centre (Centraalbureau voor Schimmelcultures) culture collection (Utrecht, The Netherlands), and permanent slides were also deposited in Herb IMI (Royal Botanic Gardens, RBG, Kew, UK). Isotype collections are held in Herbarium VIC (Universidade Federal de Viçosa, UFV, Viçosa, MG, Brazil).

### Nomenclature

The electronic version of this article in Portable Document Format (PDF) in a work with an ISSN or ISBN will represent a published work according to the International Code of Nomenclature for algae, fungi, and plants, and hence the new names contained in the electronic publication of a PLOS ONE article are effectively published under that Code from the electronic edition alone, so there is no longer any need to provide printed copies.

In addition, new names contained in this work have been submitted to MycoBank from where they will be made available to the Global Names Index. The unique MycoBank number can be resolved and the associated information viewed through any standard web browser by appending the MycoBank number contained in this publication to the prefix http://www.mycobank.org/MB/. The online version of this work is archived and available from the following digital repositories: [PubMed Central, LOCKSS, KNAW].

### Molecular characterization

#### DNA Extraction

Fungi were grown in Erlenmeyer flasks containing 100 mL of liquid medium (10 g of sucrose, 2 g L-asparagine, 2 g yeast extract, 1 g KH_2_PO_4_, 0.1 g MgSO_4_.7H_2_O, 0.44 mg ZnSO_4_.7H_2_O, 0.48 mg FeCl_3_.6H_2_O and 0.36 mg MnCl_2_.H_2_O) for 5 days at 26°C on a shaker (170 rpm). The resulting mycelium was washed out with distilled water and placed on sterile filter paper to dry. DNA extraction followed a CTAB extraction protocol modified from Doyle & Doyle [67], as follows. Using a pestle and mortar, the fungal biomass of each isolate was ground in liquid nitrogen and transferred to 1.5 mL microtubes containing 750 µL of CTAB 2× buffer and 15 µL of 2-β-mercaptoethanol. Microtubes were placed in water bath at 65°C/30 min. In each tube, 500 µL of phenol chloroform-isoamyl alcohol (25∶24∶1 v/v) was added followed by centrifugation at 14000 g/5 min. The supernatant was transferred to a new microtube and 500 µL chloroform-isoamyl alcohol (24∶1 v/v) were added; this was then centrifuged at 14000 g/5 min. An aliquot of 360 µL of the supernatant was transferred to a new tube, in which 324 µL of cold isopropyl alcohol was added. The suspension was held at −20°C for 10 min and then centrifuged at 14000 g/7 min. The supernatant was discarded and the pellet was washed twice with 500 µL ethanol 70%, followed by centrifugation at 14000 g/5 min. After the ethanol was discarded, tubes were allowed to dry at room temperature overnight. The dried pellet was then resuspended in 50 µL TE buffer containing RNAse (10 µL/mL), homogenized and held at 37°C/2 h. The quality and quantity of DNA samples were determined in agarose gels (0.8%) stained with ethidium bromide (0.15 µg/mL). A DNA mass marker λ HindIII (Invitrogen) was used in the electrophoresis at 80 V for 1 h to quantify the DNA.

#### DNA amplification and sequencing of the four new taxa

Amplification of PCR products of three genomic regions, *ITS rDNA* (Internal Transcribed Spacer regions on the nrRNA gene operon, including the intervening 5.8S nrRNA gene), *LSU rDNA* (Large Sub Unit; 28S nrRNA gene) and part of the second half of EF1-α (Elongation Factor-1 alpha) were conducted with primers ITS1-F (CTTGGTCATTTAGAGGAAGTAA) [68], ITS4-R (TCCTCCGCTTATTGATATGC) [69]; specific primers CLA-F (5′ GCATATCAATAAGCGGAGGA 3′), CLA-R (5′ GACTCCTTGGTCCGTGTTTCA 3′) [22]; and EF1-983F (5′ GCYCCYGGHCAYCGTGAYTTYAT 3′), EF1-2218R (5′ GACTTGACTTCRGTVGTGAC 3′) [28], respectively. All sequences have been deposited in GenBank under the accession numbers (JQ815076–JQ815079; JQ855712–JQ855717).

All PCR reactions were performed in a total volume of 50 µL and contained 20 to 100 ng of genomic DNA, with buffer 1× (50 mM KCl, 10 mM Tris-HCl); 1.5 mM MgCl2; 0.2 µM of each dNTP; 1 U of Taq DNA polymerase and 0.2 µM of the relevant primer. All reactions were done in a MJ Research PTC 100 thermocycler. For the ITS regions, PCR conditions were as follow: 5 min of denaturation at 95°C, followed by 30 cycles consisting of 30 s at 95°C, 30 s at 60°C and 90 s at 72°C and finally 10 min of extension at 72°C. The CLA reactions were done starting with 2 min of denaturation at 95°C, followed by 40 cycles consisting of 30 s at 95°C, 60 s at 62°C, 90 s at 72°C and finally 5 min of extension at 72°C. Meanwhile, the EF1-α reactions started with 2 min of denaturation at 95°C, followed by 40 cycles consisting of 30 s at 95°C, 60 s at 60°C and 90 s at 72°C and then 5 min of extension at 72°C. PCR products were then purified using minicolumns according to the manufacturer's protocols (Roche-High Pure PCR Product Purification Kit).

#### DNA amplification and sequencing of Escovopsis weberi (ATCC 64542) and Escovopsis aspergilloides (CBS 423.93)

Amplification of PCR products of three genomic regions, ITS, LSU and part of the first half of EF1-α were conducted with primers ITS5 (5′ GGAAGTAAAGTCGTAACAAGG 3′) [69], ITS4 (5′ TCCTCCGCTTATTGATATGC 3′) [69]; primers LR0R (5′ GTACCCGCTGAACTTAAGC 3′) [70], LR5 (5′ TCCTGAGGGAAACTTCG 3′) and EF1-728F (5′ CATCGAGAAGTTCGAGAAGG 3′) [71], EF-2 (5′ GGARGTACCAGTSATCATGTT 3′) [72], respectively. All sequences have been deposited in GenBank under the accession numbers (KF293281–KF293287, KF293275–KF293277). PCR reactions were performed in a total volume of 12.5 µL and contained 20 to 100 ng of genomic DNA, with reaction buffer 1× (Bioline); 2 mM MgCl_2_; 0.1 µM of each dNTP; 0.5 U of BioTaq DNA polymerase and 0.2 µM of the relevant primer. PCR conditions were as follow: 5 min of denaturation at 94°C, followed by 40 cycles consisting of 45 s at 94°C, 30 s at 48°C and 90 s at 72°C and finally 6 min of extension at 72°C. The annealing temperature was increased to 52°C for EF1-α. Amplification products were sequenced directly without post-PCR cleanup.

Sequencing for all strains was carried out directly from purified PCR-amplified products using the automatic sequencer ABI Prism 3100. In order to determine the generic placement of the *Escovopsis*-like isolates among the *Hypocreales* using the LSU sequence data, additional sequences from other hypocrealean species were downloaded from GenBank (http://www.ncbi.nlm.nih.gov/nuccore). Representatives from five families (*Clavicipitaceae*, *Cordycipitaceae*, *Hypocreaceae*, *Nectriaceae*, and *Ophiocordycipitaceae*), as well as the type strain sequences of *E. weberi* and *E. aspergilloides*, were sampled for a total of 49 hypocrealean taxa, excluding one *Glomerella cingulata* isolate of the *Phyllachorales* for rooting the *Hypocreales* (see Fig. 8). GenBank accession numbers of the sequences included in the phylogenetic analyses are shown in the respective phylogenetic trees. Sources of fungal species and GenBank sequences for which novel sequences were generated in this study are presented in Table S1. The ITS data was used to test for species relationships between the obtained strains. Megablast searches using the ITS sequences revealed only distant hits with other species of *Hypocreales* (less than 90% identity) and therefore the ITS phylogeny was focused on *Escovopsis* and *Escovopsioides*.

All sequences were aligned using ClustalW (http://www.ebi.ac.uk/clustalw) [73] and were edited manually using *MEGA* 5 [74].

#### Phylogenetic analyses

Molecular phylogenies of LSU (Fig. 8) and ITS (Fig. 9) were used to describe and infer the relationship among *Escovopsis* isolates collected from the Atlantic rainforest in Viçosa, Minas Gerais. To generate the EF1-α phylogenies, we started by downloading all sequences from GenBank matching the keywords “*Escovopsis*” and “elongation”. These were subsequently subjected to a multiple alignment and non-overlapping, short or “low quality” sequences were excluded. Some sequences in the GenBank accession series EF589910–EF589949 were excluded as they contained numerous N's. Based on the LSU phylogeny, related genera representing several hypocrealean families were selected and EF1-α sequences spanning the same target region identified in the *Escovopsis* alignment were downloaded where these were available, resulting in an alignment of 716 characters. The EF1-α sequences of the novel species described in this study were added to this alignment file. There is about a 373 bp overlap between the sequences in this alignment and the sequences generated for the novel *Escovopsis* species described in this study and therefore two phylogenies were derived from this alignment, the first using 716 characters but excluding the novel species (resulting in Fig. S4) and the second using 373 characters but including the novel species (resulting in Fig. S5). Maximum parsimony (MP) and maximum likelihood (ML) analyses were conducted for ITS and LSU and MP and distance (neighbor-joining with the HKY85 substitution model) analyses for EF1-α using PAUP 4.0b10 [75]; Bayesian analyses were conducted for ITS and LSU using MrBayes 3.2.1 [76]. For MP, heuristic searches with 1,000 random-addition sequence replicates and TBR (tree-bisection—reconnection) branch swapping were performed. Heuristic MP bootstrap analysis consisted of 1,000 pseudoreplicates (TBR branch swapping), with 10 random-taxon-addition replicates per pseudoreplicate.

For ML analysis, DNA sequence evolution model was established based on the Akaike information criterion (AIC) and likelihood ratio test implemented in ModelTest 3.7 [77]. Heuristic ML bootstrap analysis consisted of 100 pseudoreplicates (TBR branch swapping). The DNA substitution model was determined based on the AIC criterion of MrModelTest [78]. The GTR+I+G model (general time reversible with a proportion of sites invariant and inverse gamma-distributed rates) was used for the LSU Bayesian analysis and GTR+G model (general time reversible with gamma-distributed rates) was used for the ITS Bayesian analysis. A parallel run, each consisting of four chains, was subjected to Markov Chain Monte Carlo (MCMC) analysis until the runs converged with a split frequency of <0.01. The first 25% of the generations were discarded as “burn-in”. The MCMC analysis started with a heating parameter 0.1 from a random tree topology and lasted 1,000,000 generations (LSU) and 30,000 generations (ITS). The 50% majority consensus rule trees and posterior probabilities were calculated from 15,002 trees (LSU; Fig. 8) and 452 trees (ITS; Fig. 9). For the parsimony analysis of the LSU and EF1-α alignments, only the first 1000 equally most parsimonious trees were retained; for the ITS alignment only a single most parsimonious tree was found.

## Supporting Information

Figure S1 ***Escovopsioides nivea*** **.** (**A–D**) Details of spore forms in paratype variant that initially produced only the chlamydospore sensu lato stage (**D**); Other spore forms develop intermittently, often producing solitary, lateral phialides (**A**, arrow). All scale bars = 10 µm.
(TIF)

Figure S2 **Images supporting hypotheses relating to fungal transmission.** (**A**)–(**B**) **Structures found in ant-fungal gardens**: (**A**) Ropes of iridescent chlamydospores sensu lato of *Escovopsioides nivea* over-growing the fungal garden of an abandoned attine nest; (**B**) Close-up of chlamydospores sensu lato of *Escovopsioides nivea* within garden; (**C**) **Endophytic ability**: *Escovopsis microspora* emerging from surface-sterilized leaf of privet (*Ligustrum* sp., Oleaceae)—after 7 days on tap water agar—inoculated 2-months previously with conidia of *E. microspora* (**B–C**, scale bar = 20 µm); (**D**) Gongylidia of *Leucoagaricus* symbiont within garden.
(TIF)

Figure S3 **Fungal sampling, Mata do Paraíso, Viçosa, Minas Gerais, Brazil.** (**A**) Sampling of *Acromyrmex* nest—note, to the right, the mattock used for excavation and the nest entrance (arrow)—to expose the healthy (**B**) or diseased (**C**) fungal garden; (**D**) Close-up of ‘infected’ middens with blooms of *Escovopsis microspora* around periphery (arrows), inset (**E**) with detail of sporulation onto surrounding litter.
(TIF)

Figure S4 **Distance analysis using neighbor joining with the HKY85 substitution model on 264 aligned EF1-α sequences spanning 716 characters downloaded from GenBank.** A parsimony analysis on the same dataset was limited to 1000 equally most parsimonious trees and the derived consensus tree had the same overall topology for the terminal clades compared to the presented distance tree (the distance tree, the first of the 1000 equally most parsimonious trees and the consensus parsimony tree are available in TreeBASE). However, the more basal nodes and the overall backbone structure changed between the different analyses. In both of these analyses, lineages within families were interspersed between families and therefore the clustering presented here is not an artifact of the analysis algorithm. Distance-based bootstrap support values are shown at the nodes and thickened branches indicate the parsimony bootstrap support values (green for values >84% and blue for 70–84%).
(RAR)

Figure S5 **Distance analysis using neighbor joining with the HKY85 substitution model on 267 aligned EF1-α sequences spanning 373 characters downloaded from GenBank and including the novel species (in bold face) described in this study.** A parsimony analysis on the same dataset was limited to 1000 equally most parsimonious trees and the derived consensus tree had the same overall topology for the terminal clades compared to the presented distance tree (the distance tree, the first of the 1000 equally most parsimonious trees and the consensus parsimony tree are available in TreeBASE). However, the more basal nodes and the overall backbone structure changed between the different analyses. In both of these analyses, lineages within families were interspersed between families and therefore the clustering presented here is not an artifact of the analysis algorithm. Distance-based bootstrap support values are shown at the nodes and thickened branches indicate the parsimony bootstrap support values (green for values >84% and blue for 70–84%).
(RAR)

Table S1 Sources of fungal species and GenBank sequences for which novel sequences were generated in this study.
(PDF)

Table S2 Number of *Escovopsis* and *Escovopsioides* species isolated from individual *Acromyrmex* colonies. MB = Mata da Biologia (UFV campus); Rep = Represa UFV (UFV campus); MP = Mata do Paraíso.
(PDF)
