# Supplementary figures and images for: Yet More “Weeds” in the Garden: Fungal Novelties from Nests of Leaf-Cutting Ants
Source: PLoS One. 2013 Dec 20;8(12):e82265. doi: 10.1371/journal.pone.0082265 (PMC3869688; doi:10.1371/journal.pone.0082265)

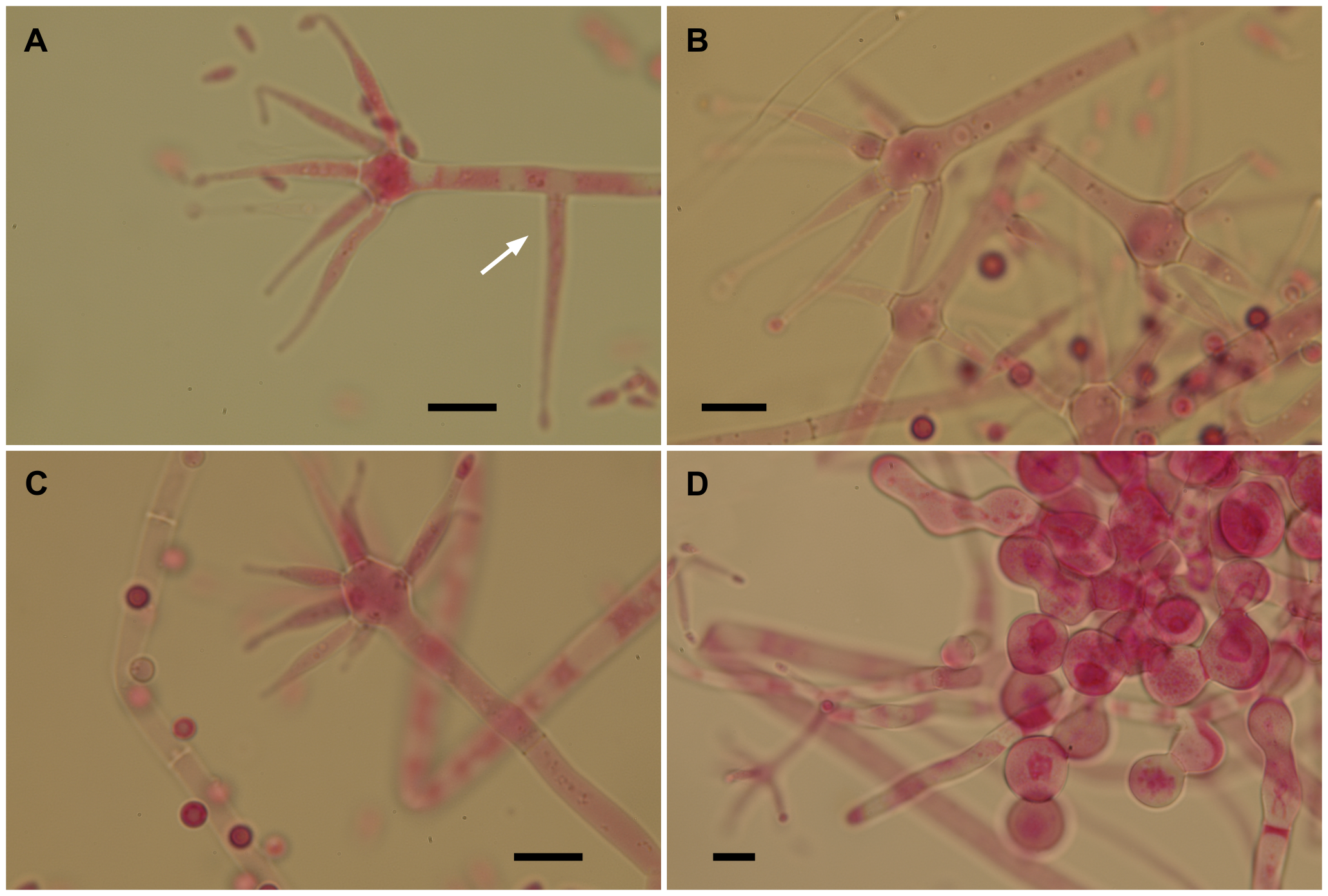

Supplement: Figure S1 — Escovopsioides nivea . (A–D) Details of spore forms in paratype variant that initially produced only the chlamydospore sensu lato stage (D); Other spore forms develop intermittently, often producing solitary, lateral phialides (A, arrow). All scale bars = 10 µm. (TIF) [file pone.0082265.s001.tif]

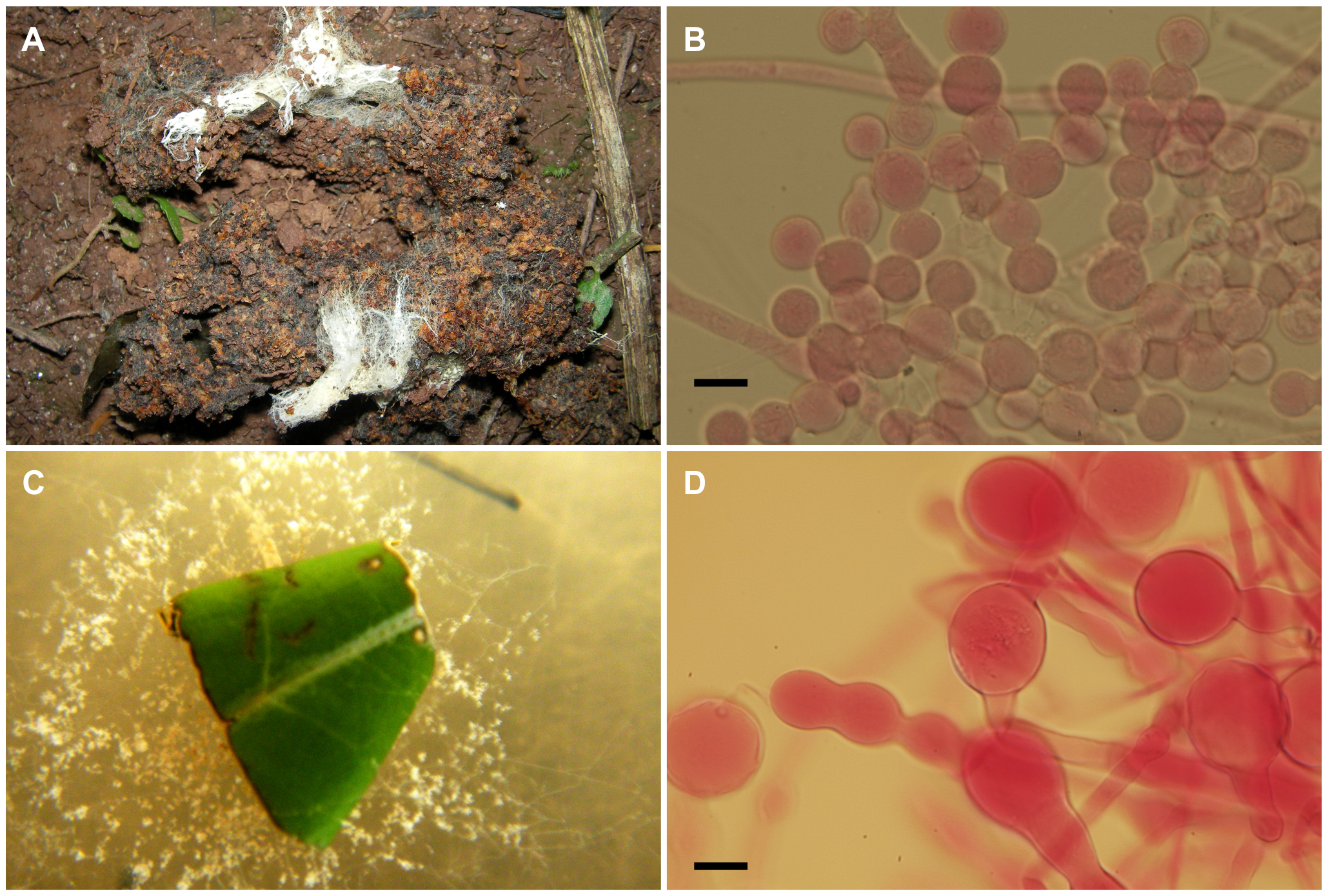

Supplement: Figure S2 — Images supporting hypotheses relating to fungal transmission. (A)–(B) Structures found in ant-fungal gardens: (A) Ropes of iridescent chlamydospores sensu lato of Escovopsioides nivea over-growing the fungal garden of an abandoned attine nest; (B) Close-up of chlamydospores sensu lato of Escovopsioides nivea within garden; (C) Endophytic ability: Escovopsis microspora emerging from surface-sterilized leaf of privet (Ligustrum sp., Oleaceae)—after 7 days on tap water agar—inoculated 2-months previously with conidia of E. microspora (B–C, scale bar = 20 µm); (D) Gongylidia of Leucoagaricus symbiont within garden. (TIF) [file pone.0082265.s002.tif]

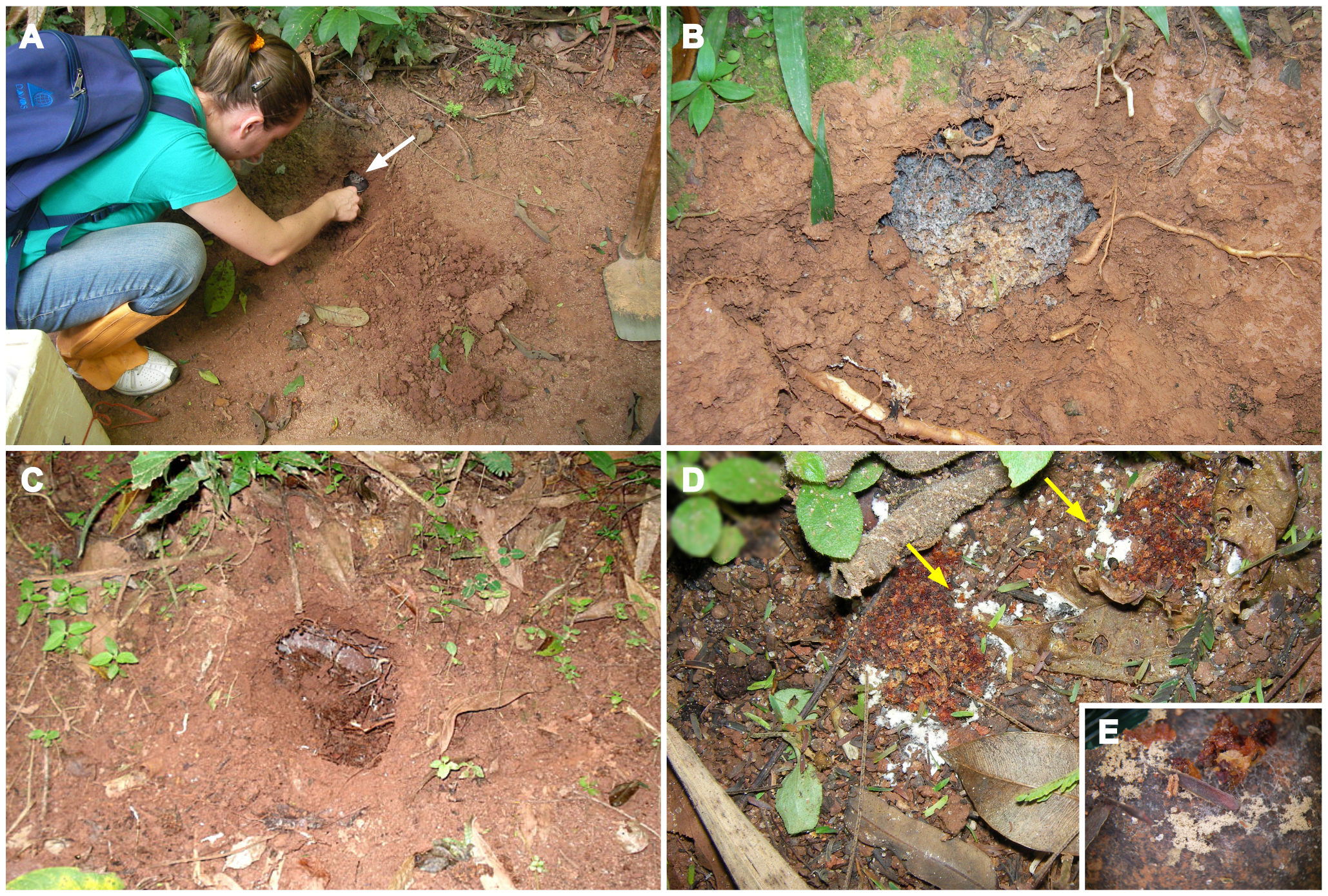

Supplement: Figure S3 — Fungal sampling, Mata do Paraíso, Viçosa, Minas Gerais, Brazil. (A) Sampling of Acromyrmex nest—note, to the right, the mattock used for excavation and the nest entrance (arrow)—to expose the healthy (B) or diseased (C) fungal garden; (D) Close-up of ‘infected’ middens with blooms of Escovopsis microspora around periphery (arrows), inset (E) with detail of sporulation onto surrounding litter. (TIF) [file pone.0082265.s003.tif]
