# Supplementary material for: Yet More “Weeds” in the Garden: Fungal Novelties from Nests of Leaf-Cutting Ants
Source: PLoS One. 2013 Dec 20;8(12):e82265. doi: 10.1371/journal.pone.0082265 (PMC3869688; doi:10.1371/journal.pone.0082265)
Supplement: Table S1 — Sources of fungal species and GenBank sequences for which novel sequences were generated in this study. (PDF) [file pone.0082265.s006.pdf]

Table S1. Sources of fungal species and GenBank sequences for which novel sequences were generated in this study.

Species	Culture number	Origin	Host / substrate	GenBank Accession Number		
				ITS	LSU	EF1-α
Escovopsioides nivea	CBS 135749, J6	Brazil: Minas Gerais	*Acromyrmex subterraneus subterraneus* fungal garden	JQ815078	JQ855716	JQ855713
Escovopsis aspergilloides	CBS 423.93, DAOM 216382	Trinidad and Tobago: Trinidad	Nest of *Trachymyrmex ruthae*	KF293287	KF293283	KF293277
Escovopsis lentecrescens	CBS 135750, J9	Brazil: Minas Gerais	*Acromyrmex subterraneus subterraneus* fungal garden	JQ815079	JQ855717	JQ855714
Escovopsis microspora	CBS 135751, J10	Brazil: Minas Gerais	*Acromyrmex subterraneus molestans* fungal garden	JQ815076	KF293284	—
Escovopsis moelleri	CBS 135748, J5	Brazil: Minas Gerais	*Acromyrmex subterraneus molestans* fungal garden	JQ815077	JQ855715	JQ855712
Escovopsis weberi	ATCC 64542	Brazil: Minas Gerais	'Carpenter ant' fungal mass	KF293285	KF293281	KF293275
Escovopsis weberi	CBS 810.71	Brazil	Ant nest	KF293286	KF293282	KF293276
