# Supplementary material for: Yet More “Weeds” in the Garden: Fungal Novelties from Nests of Leaf-Cutting Ants
Source: PLoS One. 2013 Dec 20;8(12):e82265. doi: 10.1371/journal.pone.0082265 (PMC3869688; doi:10.1371/journal.pone.0082265)
Supplement: Table S2 — Number of Escovopsis and Escovopsioides species isolated from individual Acromyrmex colonies. MB = Mata da Biologia (UFV campus); Rep = Represa UFV (UFV campus); MP = Mata do Paraíso. (PDF) [file pone.0082265.s007.pdf]

Table S2. Number of *Escovopsis* and *Escovopsioides* species isolated from individual *Acromyrmex* colonies. MB = Mata da Biologia (UFV campus); Rep = Represa UFV (UFV campus); MP = Mata do Paraíso.

Colony identity	Mutualistic fungus garden		Total
	Top	Bottom	
A. niger 1 (MB)	-	*E. nivea*<br>*E. microspora*	2
A. niger 2 (MP)	*E. nivea*	-	1
A. subterraneus molestans 1 (MB)	-	-	0
A. subterraneus molestans 2 (MB)	-	-	0
A. subterraneus molestans 3 (MB)	*E. nivea*	*E. moelleri*<br>*E. nivea*<br>*E. microspora*	3
A. subterraneus molestans 4 (MB)	*E. moelleri*<br>*E. microspora*	*E. moelleri*<br>*E. nivea*<br>*E. lentecrescens*	4
A. subterraneus molestans 5 (Rep)	*E. microspora*	*E. microspora*	1
A. subterraneus molestans 6 (Rep)	-	*E. microspora*	1
A. subterraneus molestans 7 (Rep)	-	*E. nivea*	1
A. subterraneus molestans 8 (Rep)	*E. microspora*	-	1
A. subterraneus molestans 9 (Rep)	-	*E. nivea*	1
A. subterraneus molestans 10 (MP)	-	*E. moelleri*<br>*E. microspora*	2
A. subterraneus molestans 11 (MP)	*E. nivea*	*E. moelleri*<br>*E. microspora*	3
A. subterraneus subterraneus 1 (MB)	*E. nivea*	-	1
A. subterraneus subterraneus 2 (MB)	-	-	0
A. subterraneus subterraneus 3 (MB)	-	*E. nivea*	1
A. subterraneus subterraneus 4 (MB)	-	*E. moelleri*	1
A. subterraneus subterraneus 5 (Rep)	-	*E. nivea*	1
A. subterraneus subterraneus 6 (Rep)	-	*E. microspora*	1
A. subterraneus subterraneus 7 (Rep)	*E. nivea*	-	1
A. subterraneus subterraneus 8 (MP)	-	-	0
A. subterraneus subterraneus 9 (MP)	-	*E. nivea*	1
A. subterraneus subterraneus 10 (MP)	*E. nivea*	*E. nivea*	1
A. subterraneus subterraneus 11 (MP)	*E. lentecrescens*	*E. nivea*<br>*E. lentecrescens*	2
A. subterraneus subterraneus 12 (MP)	-	*E. microspora*	1
